# The complete mitochondrial genome of the oleaginous microalgae *Ankistrodesmus falcatus* strain UCP001 from the Peruvian Amazon

**DOI:** 10.1080/23802359.2020.1846470

**Published:** 2021-01-08

**Authors:** Marianela Cobos, Gad E. Grandez, J. Dylan Maddox, Carlos G. Castro, Hicler N. Rodríguez, Segundo L. Estela, Miguel A. Grández, Jae D. Paredes, Rodil Tello-Espinoza, Pedro M. Adrianzén, Jorge L. Marapara, Juan C. Castro

**Affiliations:** a Laboratorio de Biotecnología y Bioenergética (LBB), Universidad Científica del Perú (UCP), Loreto, Perú; b Unidad Especializada de Biotecnología, Centro de Investigaciones de Recursos Naturales de la Amazonía (CIRNA), Universidad Nacional de la Amazonia Peruana (UNAP), Loreto, Perú; c Pritzker Laboratory for Molecular Systematics and Evolution, Field Museum of Natural History, Chicago, IL, USA; d Environmental Sciences, American Public University System, Charles Town, WV, USA; e Departamento Académico de Manejo Forestal y Medio Ambiente, Facultad de Ciencias Forestales, Universidad Nacional de la Amazonia Peruana (UNAP), Ciudad Universitaria - Zungarococha, Loreto, Perú; f Departamento Académico de Ciencias Biomédicas y Biotecnología, Facultad de Ciencias Biológicas, Universidad Nacional de la Amazonia Peruana (UNAP), Ciudad Universitaria - Zungarococha, Loreto, Perú

**Keywords:** Biofuel, chlorophyta, green algae, mitochondrial genome, molecular sequence annotation

## Abstract

*Ankistrodesmus falcatus* strain UCP001 is a native oleaginous microalgae isolated from the Peruvian Amazon basin. In this study we sequenced, *de novo* assembled, and functionally annotated the complete mitochondrial genome of the native oleaginous microalgae *Ankistrodesmus falcatus* strain UCP001 (Accesion number MT701044). This mitogenome is a typical circular double stranded DNA molecule of 41,048 bp in total length with G + C content of 37.4%. The mitogenome contains 49 genes, including 18 protein coding genes, 5 ribosomal (rRNA) genes and 26 transfer RNA (tRNA) genes. A phylogenetic analysis of 18 microalgae species indicated that *Ankistrodesmus falcatus* strain UCP001 was closely related to *Ourococcus multisporus* and *Raphidocelis subcapitata*. The complete mitochondrial genome sequence of *Ankistrodesmus falcatus* strain UCP001 enriches genomic resources of oleaginous native microalgae from the Peruvian Amazon for further basic and applied research.

*Ankistrodesmus falcatus* (Corda) Ralfs, 1848 strain UCP001 is a unicellular green microalgae of the Selenastraceae family isolated from the Itaya River (Cobos et al. 2017), which is a blackwater tributary river of the Amazon River in the northern region of Peru. This microalgae strain has shown a great capability to biosynthesize and store lipids (∼264 mg/g of dry biomass) under nutritive stress (Ruiz et al. 2016). To date, however, genomic resources for this microalgae strain are scarce and the elucidation of their complete mitogenome will enhance our current understanding of the structure and organization of mitogenomes in green microalgae. In addition, the genomic sequence obtained would help to define, more precisely, the phylogenetic relationships among green microalgae. Finally, this genetic information could prove useful in the development of mitochondrial vector systems for biotechnology applications. Consequently, we sequenced, *de novo* assembled, and functionally annotated the complete mitochondrial genome of the oleaginous native microalgae *Ankistrodesmus falcatus* (Corda) Ralfs, 1848 strain UCP001.

*Ankistrodesmus falcatus* (Corda) Ralfs, 1848 strain UCP001 was provided by the Peruvian Amazon Microalgae Culture Collection, Universidad Científica del Perú and cultured in CHU-10 medium until exponential growth phase. Microalgae cells were then harvested by centrifugation (5000 × g, 4 °C × 10 min), and total genomic DNA was purified using a standardized protocol. Libraries were constructed and sequenced with both PacBio RSII and Illumina HiSeq 2500 sequencing platforms. Low quality reads were removed with Trimmomatic v 0.38.0 (Bolger et al. 2014) and then filtered high quality reads, were de novo assembled with NOVOPlasty software v 4.0 (Dierckxsens et al. 2017). The complete circular mitogenome was annotated using a combination of the following bioinformatic tools: DOGMA (Wyman et al. 2004), GeSeq v 1.82 (Tillich et al. 2017), Mitofy v 3.0 (Alverson et al. 2010), tRNAScan-SE v 2.0 (Lowe and Chan 2016; Chan and Lowe 2019), and RNAweasel (Lang et al. 2007).

The complete circular mitogenome (Accession number MT701044) has a total length of 41,048 bp and G + C content of 37.4% that, contains 49 genes, including 18 protein coding genes, 5 ribosomal (rRNA) genes and 26 transfer RNA (tRNA) genes. Most protein coding genes are involved in the mitochondrial electron transport and oxidative phosphorylation system. Also, we identified six intronic open-reading frames, encoding LAGLIDADG-2 Homing Endonucleases.

To infer the phylogenetic relationship of *Ankistrodesmus falcatus* UCP001 with other green microalgae, 17 additional mitogenomes were obtained from GenBank. In total, 13 genes (*atp6, atp9, cob, cox1, cox2, cox3, nad1, nad2, nad3, nad4, nad4L, nad5, nad6*) were extracted, aligned with MAFFT v 7.0 (Katoh et al. 2019) using default parameters, trimmed with trimAl (Capella-Gutiérrez et al. 2009) with automated mode selection, and concatenated to produce a 12,499 bp alignment. PartitionFinder2 (Lanfear et al. 2017) was used to determine the best-fit partitioning scheme and models of evolution that were then used in RAxML-NG (Kozlov et al. 2019) with 10 random and 10 parsimony-based starting trees and 1000 bootstrap replications. The phylogenetic relationship indicated that *Ankistrodesmus falcatus* strain UCP001 belongs to a strongly supported monophyletic clade with *Ourococcus multisporus* and *Raphidocelis subcapitata* (Figure 1).

**Figure 1.**
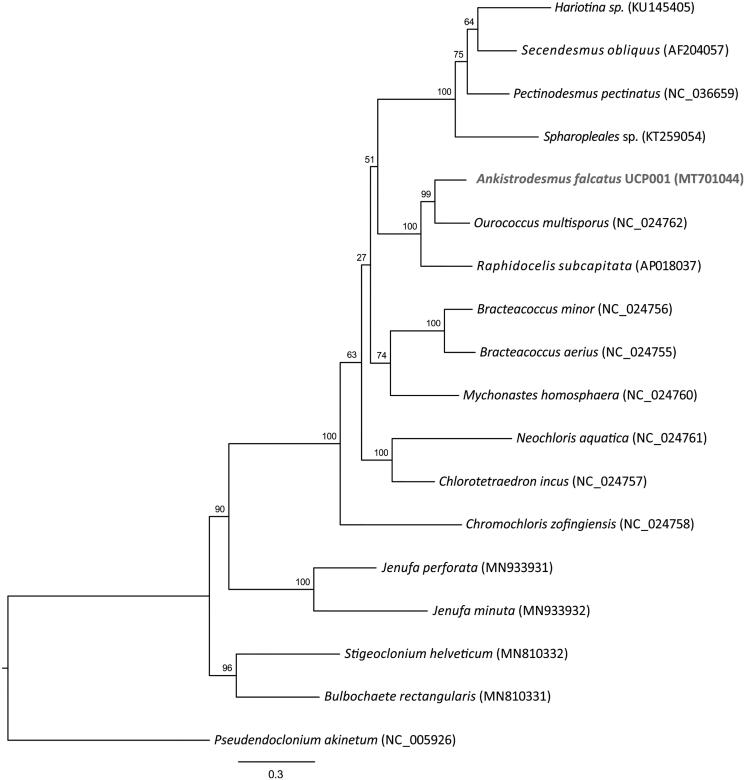
Maximum Likelihood (ML) phylogenetic tree of *Ankistrodesmus falcatus* strain UCP001 and 17 other green microalgae species based on 13 mitogenome proteins. Numbers on nodes indicate bootstrap support values based on 1000 replications. GenBank accession numbers are provided parenthetically.

## Data Availability

Mitogenome data supporting this study are openly available in GenBank at: https://www.ncbi.nlm.nih.gov/nuccore/MT701044. Associated BioProject, SRA, and BioSample accession numbers are https://www.ncbi.nlm.nih.gov/bioproject/PRJNA628966, https://www.ncbi.nlm.nih.gov/sra/SRX9346510, and SAMN16512711, respectively.

## References

[CIT0001] Alverson AJ, Wei X, Rice DW, Stern DB, Barry K, Palmer JD. 2010. Insights into the evolution of mitochondrial genome size from complete sequences of *Citrullus lanatus* and *Cucurbita pepo* (Cucurbitaceae). Mol Biol Evol. 27(6):1436–1448. 20118192 10.1093/molbev/msq029 PMC2877997

[CIT0002] Bolger AM, Lohse M, Usadel B. 2014. Trimmomatic: a flexible trimmer for Illumina sequence data. Bioinformatics. 30(15):2114–2120. 24695404 10.1093/bioinformatics/btu170 PMC4103590

[CIT0003] Capella-Gutiérrez S, Silla-Martínez JM, Gabaldón T. 2009. trimAl: a tool for automated alignment trimming in large-scale phylogenetic analyses. Bioinforma Oxf Engl. 25(15):1972–1973. 10.1093/bioinformatics/btp348 PMC2712344 19505945

[CIT0004] Chan PP, Lowe TM. 2019. tRNAscan-SE: searching for tRNA genes in genomic sequences. Methods Mol Biol Clifton NJ. 1962:1–14. 10.1007/978-1-4939-9173-0_1 PMC6768409 31020551

[CIT0005] Cobos M, Paredes JD, Maddox JD, Vargas-Arana G, Flores L, Aguilar CP, Marapara JL, Castro JC. 2017. Isolation and characterization of native microalgae from the Peruvian Amazon with potential for biodiesel production. Energies. 10(2):224.

[CIT0006] Dierckxsens N, Mardulyn P, Smits G. 2017. NOVOPlasty: de novo assembly of organelle genomes from whole genome data. Nucleic Acids Res. 45(4):e18. 28204566 10.1093/nar/gkw955 PMC5389512

[CIT0007] Katoh K, Rozewicki J, Yamada KD. 2019. MAFFT online service: multiple sequence alignment, interactive sequence choice and visualization. Brief Bioinform. 20(4):1160–1166. 28968734 10.1093/bib/bbx108 PMC6781576

[CIT0008] Kozlov AM, Darriba D, Flouri T, Morel B, Stamatakis A. 2019. RAxML-NG: a fast, scalable and user-friendly tool for maximum likelihood phylogenetic inference. Bioinformatics. 35(21):4453–4455. 31070718 10.1093/bioinformatics/btz305 PMC6821337

[CIT0009] Lanfear R, Frandsen PB, Wright AM, Senfeld T, Calcott B. 2017. PartitionFinder 2: new methods for selecting partitioned models of evolution for molecular and morphological phylogenetic analyses. Mol Biol Evol. 34(3):772–773. 28013191 10.1093/molbev/msw260

[CIT0010] Lang BF, Laforest M-J, Burger G. 2007. Mitochondrial introns: a critical view. Trends Genet. 23(3):119–125. 17280737 10.1016/j.tig.2007.01.006

[CIT0011] Lowe TM, Chan PP. 2016. tRNAscan-SE On-line: integrating search and context for analysis of transfer RNA genes. Nucleic Acids Res. 44(W1):W54–57. 27174935 10.1093/nar/gkw413 PMC4987944

[CIT0012] Ruiz MC, Rodríguez JDP, Gómez JCC. 2016. Inducción de la producción de lípidos totales en microalgas sometidas a estrés nutritivo. Acta Biológica Colomb. 21(1):17–26.

[CIT0013] Tillich M, Lehwark P, Pellizzer T, Ulbricht-Jones ES, Fischer A, Bock R, Greiner S. 2017. GeSeq - versatile and accurate annotation of organelle genomes. Nucleic Acids Res. 45(W1):W6–W11. 28486635 10.1093/nar/gkx391 PMC5570176

[CIT0014] Wyman SK, Jansen RK, Boore JL. 2004. Automatic annotation of organellar genomes with DOGMA. Bioinformatics. 20(17):3252–3255. 15180927 10.1093/bioinformatics/bth352

